# miR-183 inhibits TGF-β1-induced apoptosis by downregulation of PDCD4 expression in human hepatocellular carcinoma cells

**DOI:** 10.1186/1471-2407-10-354

**Published:** 2010-07-06

**Authors:** Jipeng Li, Hanjiang Fu, Chengwang Xu, Yi Tie, Ruiyun Xing, Jie Zhu, Yide Qin, Zhixian Sun, Xiaofei Zheng

**Affiliations:** 1 Beijing Institute of Radiation Medicine, 27 Taiping Road, Beijing 100850, People's Republic of China; 2 Department of Biochemistry and Molecular Biology, Anhui Medical University, 81 Meishan Road, Hefei 230032, People's Republic of China

## Abstract

**Background:**

In recent years, some miRNAs have been reported to be connected closely with the development of human hepatocellular carcinoma. In our previous studies, a set of miRNAs were revealed to be dysregulated in HCC tissues. However, the functions of these miRNAs in HCC remain largely undefined.

**Methods:**

The expression profiles of miR-183 were compared between HCC tissues and adjacent normal liver tissues using qRT-PCR method. This method was used to screen the potential target genes of miR-183. A luciferase reporter assay was conducted to confirm target association. Finally, the functional effect of miR-183 in hepatoma cells was examined.

**Results:**

Among the 25 HCC samples analyzed, microRNA-183 was significantly up-regulated (twofold to 367-fold) in 17 samples compared with the matching nontumoral liver tissues. Programmed cell death 4 (PDCD4) was identified as the target gene of miR-183. Moreover, PDCD4 is a proapoptotic molecule involved in TGF-β1-induced apoptosis in human HCC cells, we found that miR-183 transfectants were resistant to apoptosis induced by TGF-β1.

**Conclusions:**

We conclude that miR-183 can inhibit apoptosis in human HCC cells by repressing the PDCD4 expression, and miR-183 may play an important role in HCC development.

## Background

Hepatocellular carcinoma (HCC) is a global health problem, with over 700,000 cases worldwide each year [1]. In the USA alone, it is estimated that there will be over 20,000 new cases of primary liver cancers in 2008, with the majority being HCC [2]. In china, HCC is the second highest cancer killer since the 1990s, which alone accounts for 53% of all liver cancer deaths worldwide [3]. Various molecular alterations occur in preneoplastic nodules and escalate in HCC [4]. Several studies have shown that specific miRNAs are aberrantly expressed in malignant HCC cells or tissues compared to non-malignant hepatocytes or tissue [5-8].

miRNAs have been discovered as naturally occurring non-coding RNAs, controlling gene expression via specific sites at the 3'-UTR of target-mRNAs, causing translational repression or degradation [9,10]. Recent evidence has shown that miRNA mutations or mis-expression correlate with various human cancers and indicated that miRNAs can function as tumour suppressors and oncogenes. For example, let-7, downregulated in lung cancer, suppresses Ras [11]. miR-15 and miR-16, deleted or downregulated in leukemia [12], suppress BCL2 [13], miR-17-5p and miR-20a control the balance of cell death and proliferation driven by the proto-oncogene c-Myc [14]. Many miRNAs such as miR-21, miR-224, miR-34a, miR-221/222, miR-106a, and miR-203 are upregulated in HCC compared to benign hepatocellular tumors such as adenomas or focal nodular hyperplasia. Many other miRNAs have been noted to be decreased in HCC compared to non-tumoral tissue, such as miR-122a, miR-422b, miR-145, and miR-199a [5-8,15].

In this study, we compared the miR-183 expression profile from HCC tumor tissues and adjacent normal liver tissues. We found that miR-183 was up-regulated in HCC tumor tissues. Sequence analysis suggested a likely interaction between the 3'-UTR of PDCD4 mRNA and miR-183. Therefore, we validated that miR-183 could repress the expression of PDCD4 and analyzed its functions in human HCC cells.

## Methods

### Patients and Tumor Characteristics

HCCs and surrounding control tissue specimens were obtained from 25 patients at General Hospital of PLA (Beijing, P.R. China) after surgical resection with informed consent. The tumor tissues and adjacent normal tissues were frozen in liquid nitrogen after resection. No patient in the current study received chemotherapy or radiation therapy before the surgery. Liver samples were fully clinically characterized (Table 1). This study was performed with the approval of the Medical Ethical Committee of General Hospital of PLA.

**Table 1:** miR-183 and PDCD4 mRNA Expression Profiles in human hepatoma carcinoma tissues

Patient No.	Age	Gender	Tumer Size (cm*cm*cm)	Edmondson Grade	HBsAg	HCV-Ab	Cirrhosis	Normalized miR-183 Amount in Tumor Tissue Relative to Adjacent Normal Tissue 2^-ΔΔCt^	Normalized PDCD4 mRNA Amount in Tumor Tissue Relative to Adjacent Normal Tissue 2^-ΔΔCt^
1	35	F	5*4*4	3	Positive	Negative	Yes	32.90	0.37
2	52	M	8.5*7*6	2	Positive	Negative	No	16.68	0.41
3	43	M	3.5*3*2	2	Positive	Negative	Yes	6.36	0.89
4	41	F	12*10*8	3	Positive	Negative	No	3.39	0.32
5	34	M	10*7*7	3	Positive	Negative	Yes	4.96	0.41
6	50	M	2.5*2.5*2	3	Positive	Negative	No	23.59	0.74
7	33	M	10*8*7	4	Positive	Negative	Yes	0.33	1.45
8	50	M	2.5*1.8*1.5	1	Negative	Negative	No	0.53	0.34
9	52	M	10.5*8*5	3	Positive	Negative	Yes	2.08	0.4
10	52	M	10.5*8*5	3	Positive	Negative	Yes	9.06	0.92
11	63	M	14*10*10	2	Positive	Negative	Yes	1.29	1.25
12	70	M	3.5*3.5*3	2	Positive	Negative	Yes	25.28	0.08
13	66	F	15*7*5	3	Positive	Negative	No	48.84	0.31
14	39	M	12*11*6	3	Positive	Negative	No	5.94	0.50
15	51	M	2.5*2*2	2	Positive	Negative	Yes	101.83	0.35
16	47	M	5*5*4	2	Positive	Negative	Yes	0.28	0.98
17	37	M	16*4*8	2	Positive	Negative	Yes	367.09	0.21
18	52	M	8*7*4.5	2	Positive	Negative	No	0.39	1.57
19	64	M	8*5*4	2	Negative	Positive	No	0.17	6.19
20	50	F	6*5.5*2.5	1	Negative	Negative	No	130.69	0.2
21	52	M	7*7*7	3	Positive	Negative	Yes	1.38	0.23
22	56	M	8*6.5*5	3	Positive	Negative	Yes	0.88	0.47
23	48	M	2*2*1.5	2	Positive	Negative	No	48.17	0.17
24	59	M	8.8*8.5*8	2	Negative	Negative	No	41.93	0.26
25	40	M	2.5*2.5*2	2	Positive	Negative	Yes	16.34	0.41

HBsAg indicates hepatitis B surface antigen; HCV-Ab, hepatitis C virus antibody; M, male; F, female; miRNA, microRNA. Relative quantification was performed by the 2^-ΔΔCt ^method with adjacent normal liver tissue sample as a calibrator. Data show the means from three independent analyzes. Every independent analyze was carried out after the RNA extraction step. ΔC_T _obtained from real-time PCR was subject to paired t test. The expression levels of miR-183 in tumor tissues were significantly higher than in adjacent normal tissues (P < 0.01, t = - 3.07), and the expression levels of PDCD4 mRNA in tumor tissues were significantly lower than in adjacent normal tissues (P < 0.01, t = 3.69)

### Cell Lines and Cultures

HepG2 and Huh7 cell lines were grown in Dulbecco's modified Eagle medium (DMEM) (GIBCO BRL, Grand Island, NY) containing 10% fetal bovine serum (FBS) with 100 μg/ml penicillin/streptomycin at 37°C with 5% CO_2_.

### Vector Construction

Wild-type 3'-untranslated region (3'-UTR) of PDCD4 containing predicted miR-183 target sites were amplified by PCR from HepG2 cell genomic DNA. Primers used: Forward: GAT CTG CAG AAG AAC TCT TGC AGT CTT AGA; Reverse: GAT CAT ATG ATG TCA AGC TTT GGG TCT CTG. Mutant 3'-UTRs were generated by overlap-extension PCR method. Both wild-type and mutant 3'-UTR fragments was subcloned into the pGL3-control vector (Promega, Madison, WI) immediately downstream of the stop codon of the luciferase gene, as described before [16]. DNA fragment coding PDCD4 protein was amplified by PCR from HepG2 cell cDNA, and cloned into pCMV-Myc expression vector (Clonetech, Mountain View, CA). Primers used: Forward: GCTG AAT TCG GAT GGA TGT AGA AAA TGA GCA GA; Reverse: CTG CTC GAG TCA GTA GCT CTC TGG TTT AAG A.

### miRNAs, siRNAs and transfection

The miRNAs, siRNAs and miRNA inhibitor were synthesized by GenePharma (Shanghai, China) (miR-183: 5'-UAU GGC ACU GGU AGA AUU CAC UG-3'/5'-GUG AAU UCU ACC AGU GCC AAA AU-3'; the si-PDCD4 target sequences were GUG UUG GCA GUA UCC UUA G [17]; miR-183 inhibitor: 5'-AGU GAA UUC UAC CAG UGC CAU A-3'). siRNAs and miRNAs transfections were performed using Lipofectamine 2000 (Invitrogen, Carlsbad, CA, USA). In brief, cells were plated in 6-well plate to 40% confluence. For each well, 5 μl siRNA or miRNA (20 μM) were added into 250 μl Opti-MEM medium, 3 μl of Lipofectamine 2000 into 250 μl Opti-MEM medium and then mixed siRNA or miRNA with Lipofectamine 2000. The mixture was added to cells and incubated for 6 h before replacing the medium. Total RNA and protein were prepared 48 h after transfection and were used for qRT-PCR or western blot analysis.

### RNA extraction and qRT-PCR

Total RNA was extracted from the cultured cells using Trizol Reagent (Invitrogen) according to the manufacturer's protocol. qRT-PCR was used to confirm the expression levels of mRNAs and miRNAs. For mRNAs detection, reverse transcription was performed according to the protocol of Improm-II™ Reverse Transcriptase System (Promega); qPCR was performed as described in the method of SYBR premix Ex Taq (TaKaRa, Dalian, China) with Mx3000p (Stratagen, La Jolla, CA, USA) supplied with analytical software. GAPDH mRNA levels were used for normalization. For miRNAs detection, the qRT-PCR was performed as described before [16]. One primer of miRNAs amplification is miRNA specific, and the other is a universal primer. U6 snRNA levels were used for normalization. The oligonucleotides used as primers was: PDCD4-F 5'-TGG ATT AAC TGT GCC AAC CA-3', PDCD4-R 5'-TCT CAA ATG CCC TTT CAT CC-3'; G3PDH-F 5'-TCA GTG GTG GAC CTG ACC TG-3', G3PDH-R 5'-TGC TGT AGC CAA ATT CGT TG-3'; miR-RT primer 5'-GCG AGC ACA GAA TTA ATA CGA CTC ACT ATA GG(T)18VN-3'; miR-183 5'-TATGGCACTGG TAGAA TTC ACT-3'; universal primer 5'-GCG AGC ACA GAA TTA ATA CGA C-3'; U6-F 5'-CGC TTC GGC AGC ACA TAT ACT A-3', U6-R 5'-CGC TTC ACG AAT TTG CGT GTC A-3'.

### Western blot analysis

Protein extracts were prepared by a modified RIPA buffer with 0.5% sodium dodecyl sulfate (SDS) in the presence of proteinase inhibitor cocktail (Complete mini, Roche, Indianapolis, IN, USA). Polyacrylamide gel electrophoresis, tank-based transfer to Immobilon Hybond-C membranes (Amersham Biosciences) and immunodetection were performed with standard techniques. Antibodies against PDCD4 (12587-1-AP, ProteinTech, Chicago, USA) and β-actin antibody (Zhongshan Biotechnology, Beijing, China) were used in western analysis in accordance with the manufacturer's instruction. Signals were visualized with SuperSignal^® ^West Pico chemoluminescent substrate (Pierce, Rockford, IL, USA) by exposure to films.

### Luciferase Reporter Assay

HepG2 cells were transfected in 24-well plates using Lipofectamine 2000 transfection reagent. The transfection mixtures contained 100 ng of firefly luciferase reporter plasmid and 5 pmol of miR-183 or 20 pmol of miR-183 inhibitor. pRL-TK (Promega) was also transfected as a normalization control. Cells were collected 48 h after transfection, and luciferase activity was measured using a dual-luciferase reporter assay system (Promega).

### Cell apoptosis

Huh7 Cells were transfected with miRNAs or siRNAs. TGF-β1 (Sigma, st. Louis, MO, USA) was added 24 h after transfection. 48 h after incubated, the cells were fixed, stained with DAPI and analyzed for morphological characteristics associated with apoptosis. Annexin-V assays were performed using Annexin V-FITC kits purchased from KeyGEN (Nanjing, china) according to manufacturer's protocols. The data are expressed as the percentage of apoptotic cells.

### Statistical Analysis of Data

Unless stressed, all results are expressed as means ± SD. Differences were assessed by two tailed Student t test using Excel software. P < 0.05 was considered to be statistically significant. Paired student's t-test was performed to determine the difference of miRNA's expression levels observed between non-cancerous and cancer tissues.

## Results

### miR-183 Is Upregulated in Human HCC

Expressions of miR-183 were examined further in an independent series of primary HCC tumors and adjacent nontumoral livers (The clinical parameter of the HCC patients was shown in Table 1) by using Real-time PCR method. The results shown that miR-183 was significantly up-regulated (2 to 300 fold) in 68% of tumors (17 of 25 patients) compared to the matching adjacent nontumoral liver tissues (Table 1). These results suggest that up-regulation of miR-183 may be involved in most of human HCC development.

### miR-183 represses the expression of PDCD4

**Figure 1:**
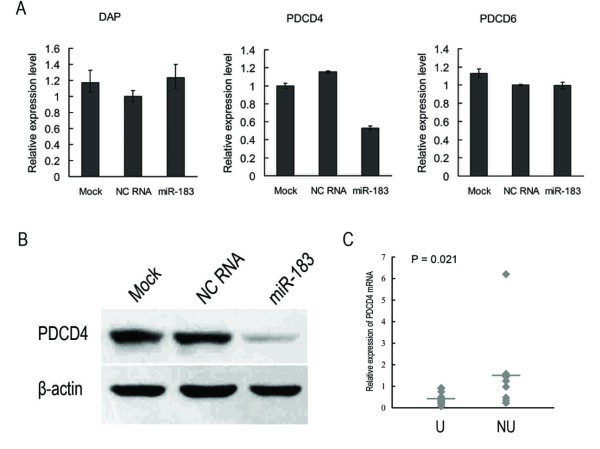
**miR-183 inhibits the mRNA and protein expression of PDCD4**. (A) HepG2 cells transfected with miR-183 compared with negative control transfected cells for 48 hours, the mRNA expression levels of three potential target genes were detected by qRT-PCR. (B) PDCD4 protein was detected by western blot using anti-PDCD4 antibody and anti-β-actin antibody as a control. (C) Correlation between expression levels of miR-183 and PDCD4. U indicates up-regulation of miR-183 in HCC tissues, and NU indicates down-regulation and no significant change of miR-183 in HCC tissues. The mean value is shown as a horizontal line. Statistical analysis was performed with Student's t-test.

To identify the functions of miR-183 in HCC cells, we analyzed putative target genes by using the TargetScan http://www.targetscan.org bioinformatics tools [18]. miR-183 maybe play the role of oncogene, considering it is up-regulated in HCC. For this reason, three tumor suppressor gene, such as death-associated protein (DAP), programmed cell death 4 (PDCD4) and programmed cell death 6 (PDCD6) were selected for further validation. To identify the genuine targets, synthetic miR-183 and negative control RNA were transfected into hepatoma Huh7 cells. qRT-PCR was used to detect the expression levels of those putative target genes. The results of qRT-PCR showed that PDCD4 was down-regulated in Huh7 cells transfected with miR-183, whereas the expression levels of other two genes had no significant change (Fig. 1A).

Western blot analysis also showed that miR-183 markedly reduced protein expression levels of PDCD4 compared with negative control RNA and mock transfected cells (Fig. 1B). All these results reveal that PDCD4 is regulated by miR-183.

To determine the clinical significance of miR-183 target genes, we examined PDCD4 mRNA levels in 25 pairs of matched HCC specimens by qRT-PCR. As expected in Table 1, lower levels of PDCD4 were detected in 17 of 25 tumor tissues as compared with the non-tumor counterparts. Furthermore, PDCD4 mRNA levels were inversely correlated with miR-183 expression (Fig. 1C), suggesting that the decreased PDCD4 expression might result from a high expression of miR-183 in HCC. However, we also observed that miR-183 up-regulation in #3, 6, 10, 14 and down-regulation in #8, 16 could not account for the expression levels of PDCD4. These data suggest that miR-183 might play a critical role on PDCD4 regulation in over a half but not all of the HCC patients. These might also represent the complexity of PDCD4 regulation especially in HCC. We speculate that other factors (either protein or RNA) might antagonize or interferer the effect of miR-183 on PDCD4, which needs further investigations.

### Interaction of miR-183 with the 3'-UTR of the PDCD4 mRNA

**Figure 2:**
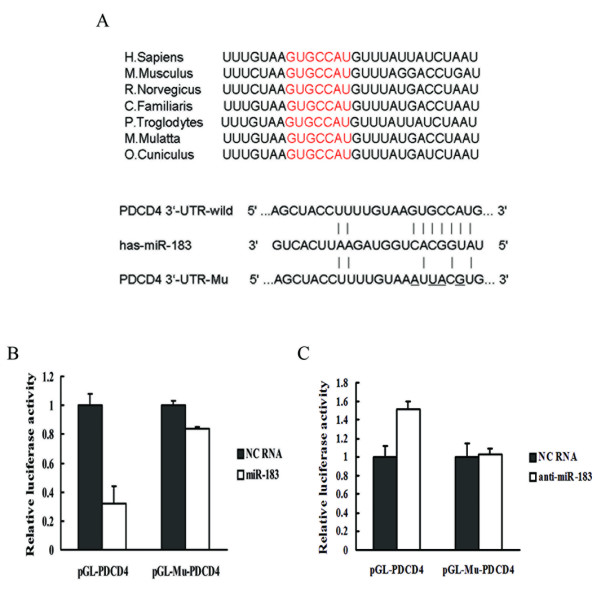
**miR-183 can act on the 3'-UTR of PDCD4 directly**. (A) The target site of miR-183 in PDCD4 3'UTR is conserved (shown in red) in many species. The PDCD4 3'-UTR mutant is identical to the wild-type, except that it has four point substitutions (red) disrupting pairing to miR-183 seed. (B) and (C) Luciferase assays indicated that miR-183 downregulated the expression of PDCD4 by targeting putative target site. NC RNA, miR-183, and miR-183 inhibitor were cotransfected with a modified pGL-3 control vector containing wild-type PDCD4 3'-UTR or mutant, respectively.

Using bioinformatic analysis, we found that the putative miR-183 binding site of PDCD4 3'-UTR was conserved across various species (Fig. 2A). To determine whether PDCD4 was direct targets of miR-183, the human PDCD4 wild-type-3'-UTR containing the miR-183 binding site was cloned into a modified pGL-3 control vector. In parallel, we constructed another reporter vector containing the mutated PDCD4 3'-UTR sequence. These luciferase reporter vectors were cotransfected into HepG2 cells with negative control RNA or miR-183 respectively. The results of luciferase assays revealed that miR-183 could act on wild type PDCD4 3'-UTR, and led to a significant decrease of luciferase activity as compared to control RNA (Fig. 2B). When the putative miR-183 binding site was mutated, there was no significant decrease in relative luciferase activity compared with control RNA (Fig. 2B). The corresponding results were observed when the expression of miR-183 was repressed by antisense RNA (Fig. 2C). These data suggest miR-183 may repress the expression of PDCD4 at posttranscriptional level by targeting its 3'-UTRs. In other words, PDCD4 is a direct target gene of miR-183.

### miR-183 inhibits TGF-β1-induced apoptosis in Huh7 cells

**Figure 3:**
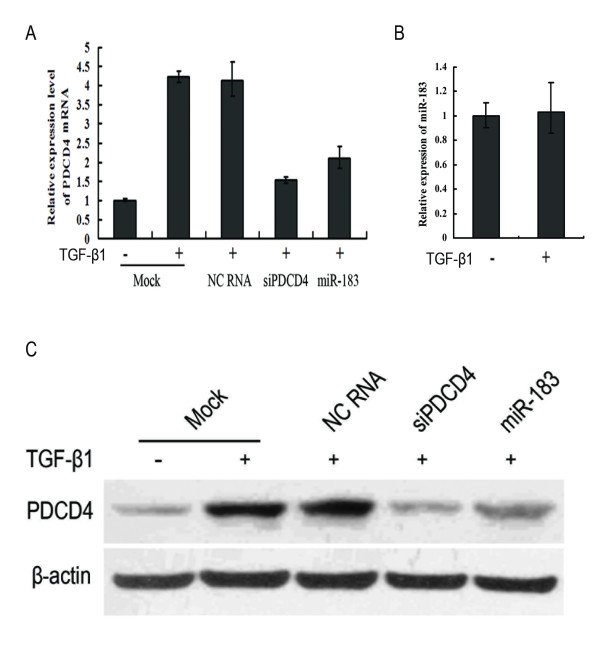
**miR-183 inhibits TGF-β1-induced PDCD4 expression in Huh7 cells**. Cells were transfected with NC RNA, siPDCD4, or miR-183 respectively, after 24 hours, TGF-β1 (5 ng/mL) was added. Cells were harvested 24 hours later, and were subsequently processed for qRT-PCR (A) and Western blot (C) analysis by hybridization with antibodies against PDCD4 and β-actin. At the same time, the effect of TGF-β1 on miR-183 was examined (B). Huh7 cells were treated with TGF-β1 (5 ng/mL) for 48 h, then harvested and processed for qRT-PCR analysis.

PDCD4 is down-regulated in human HCC tissues compared to corresponding non-tumoral liver tissues. Furthermore, PDCD4 is involved in TGF-β1 induced apoptotic signaling pathways in the HCC cell line Huh7 [19]. To investigate the function of miR-183 in HCC cells, we transfected Huh7 cells with negative control RNA, PDCD4 siRNA and miR-183 respectively, TGF-β1 was added 24 h after transfection, and cells were further incubated for 24 h. qRT-PCR and western blot results showed that miR-183 and siRNA against PDCD4 led to in a significant decrease of endogenous PDCD4 mRNA and protein levels compared to mock or negative control transfection in Huh7 cells (Fig. 3A, C). At the same time, we found that there were no significant changes in miR-183 expression levels between control cells and Huh7 cells treated with TGF-β1 (Fig. 3B).

**Figure 4:**
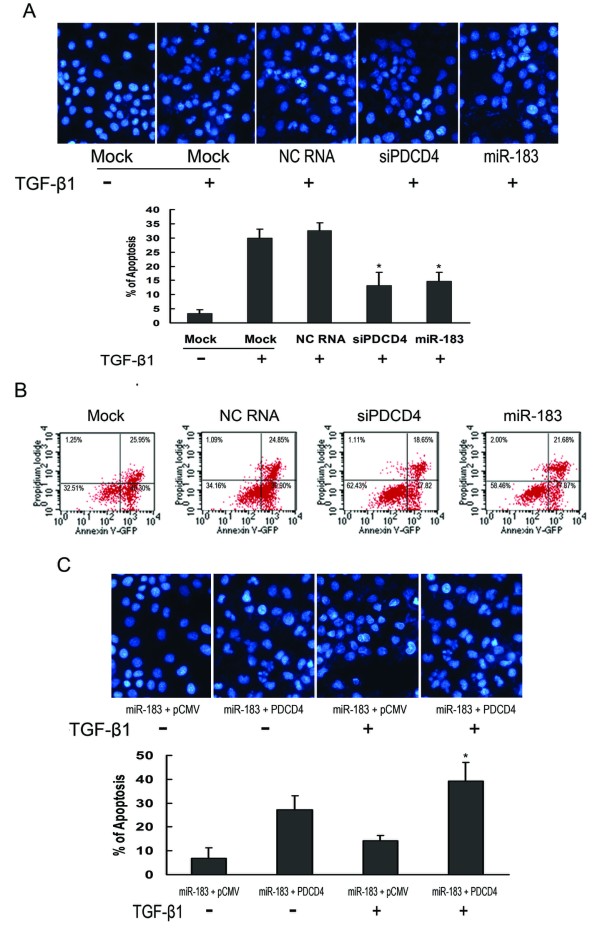
**miR-183 transfectants were resistant to apoptosis induced by TGF-β1**. (A) DAPI assay. Huh7 cells were transfected with NC RNA, siPDCD4, or miR-183 respectively, after 24 hours, TGF-β1 (5 ng/mL) was added. Cells were further incubated for 48 hours, fixed, and stained with DAPI. *P < 0.05 compared with Huh7 cells transfected with NC RNA. (B) Annexin-V assays. Huh7 cells were transfected and cultured as described in (A), then collected by trypsinization followed by centrifugation and stained with FITC conjugated Annexin-V and PI. (C) miR-183 and PDCD4 expression vector were cotransfected into Huh7 cells, after 24 hours, TGF-β1 (5 ng/mL) was added. Cells were further incubated for 48 hours, fixed, and stained with DAPI. * P < 0.05 compared with Huh7 cells transfected with control pCMV vector.

PDCD4 is a proapoptotic molecule involved in TGF-β1-induced apoptosis in human HCC cells [19], and miR-183 can inhibit TGF-β1 induced PDCD4 expression in Huh7 cells (Fig. 3A, C). Therefore, we wondered whether miR-183 could influence apoptosis of Huh7 cells. To eliminate the off-target effect of miR-183 on apoptosis, we used siRNA to down-regulate PDCD4 gene expression. Both of DAPI staining of cells (Fig. 4A) and Annexin-V assays (Fig. 4B) showed that the number of apoptotic cells induced by TGF-β1 treatment was reduced in the miR-183 and si-PDCD4 transfectants as compared to the mock and negative control cells. To finalize the function of miR-183-targeting these genes in apoptosis regulation, rescue experiments were carried out. Utilizing pCMV-Myc expression vector, PDCD4 construct without wild 3'-UTR was generated. As shown in Fig. 4C, the inhibition effect of miR-183 on apoptosis was rescued when PDCD4 expression vector was cotransfected. These results indicated that miR-183 was resistant to TGF-β1-induced apoptosis of Huh7 cells.

## Discussion

In the current study, we show that miR-183 expression is up-regulated in human HCC compared with matching adjacent nontumoral tissue. We also show that PDCD4 is negatively regulated by miR-183 at the posttranscriptional level, via a specific target site within the 3'^-^UTR. Moreover, we demonstrated that miR-183 was resistant to TGF-β1-induced apoptosis of Huh7 cells, via repression of PDCD4 expression. These results support an essential role of miR-183 in HCC development.

miR-183 is one member of miR-182-183 miRNA cluster located in the 7q31-34 locus [20]. This miRNA cluster including three miRNAs, i.e., miR-96, miR-182 and miR-183. These miRNAs share highly homologous 5'-seed sequences. Motoyama et al. have found an overexpression of miR-183 in human colorectal cancer and Lin et al. have reported that the miR-183-96-182 cluster was frequently amplified in melanoma [21,22]. Here, we found that miR-183 was resistant to TGF-β1-induced apoptosis in Huh7 cells, via repression of PDCD4 expression. Thus, it is likely that miR-183 acts as an oncogene in a variety of tumor types. However, miR-183 was identified as a potential metastasis-inhibitor in lung cancer cells [23]. These data suggest that the effect of miR-183 as an oncogene is cell type dependent.

The programmed cell death 4 (PDCD4) was originally identified as a tumor-related gene in humans and mapped to chromosome10q24 [19,24]. PDCD4 protein is known to bind eukaryotic initiation factor 4A (eIF4A), inhibit translation initiation [25,26]. Moreover, PDCD4 has been found to inhibit AP-1-mediated trans-activation and to induce expression of the cyclin-dependent kinase inhibitor p21. As a result, loss of PDCD4 confers growth advantages to the cells by several means and thereby facilitates the development of cancer. PDCD4 expression is down-regulated or lost in several tumor types [27]. However, relatively little is known about mechanisms regulating PDCD4 expression in cancer cells. Initial studies have suggested that PDCD4 is regulated by topoisomerase-inhibitors, COX-2-inhibitors, Myb and Akt [28]. In a recent study, PDCD4 has been reported as a functional target of miR-21 in various aspects of tumor progression: cell proliferation, invasion, metastasis, and neoplastic transformation in breast cancer [27,29,30], invasion, intravasation, and metastasis in colon cancer [28], proliferation and invasion in esophageal squamous cell carcinoma [31]. Here, we found that PDCD4 was negatively regulated by miR-183 in HCC cells. All these data suggest the regulation mechanism of PDCD4 is very complex, many factors (either protein or RNA) might influence the expression of PDCD4.

## Conclusions

This study suggests that miR-183, up-regulated in HCC, represses the expression of the tumor-suppressor PDCD4 posttranscriptionally, and inhibits TGF-β1-induced apoptosis in human HCC cells. Therefore, miR-183 may play an important role in HCC development.

## Abbreviations

miRNA: microRNA; PDCD4: Programmed cell death protein 4; UTR: untranslated region; qRT-PCR: quantitative real-time PCR.

## Competing interests

The authors declare that they have no competing interests.

## Authors' contributions

JL, HF, CX, YT, RX and JZ performed experiments; HF and XZ designed research and wrote the paper; HF, YQ, ZS and XZ analyzed data. All authors read and approved the final manuscript.

## Pre-publication history

The pre-publication history for this paper can be accessed here:

http://www.biomedcentral.com/1471-2407/10/354/prepub
